# Supplementary material for: Characterization of Inflammasomes and Their Regulation in the Red Fox
Source: Animals (Basel). 2023 Dec 13;13(24):3842. doi: 10.3390/ani13243842 (PMC10741141; doi:10.3390/ani13243842)
Supplement: Supplementary file 1 [file animals-13-03842-s001.zip › animals-2768120-supplementary.pdf]

Supplementary Information

Characterization of inflammasomes and their regulation in the red fox

Huijeong Ahn[1†], Dong-Hyuk Jeong[2†], Gilyoung Lee[1], Suk-Jin Lee[3], Jeong-Jin Yang[3], Yo-Han Kim[1], Tae-Wook Hahn[1], Sooyoung Choi[1], and Geun-Shik Lee[1*]

[1]College of Veterinary Medicine and Institute of Veterinary Science, Kangwon National University, Chuncheon, 24341, Republic of Korea.

[2]Laboratory of Wildlife Medicine, College of Veterinary Medicine, Chungbuk National University, Cheongju, 28644, Republic of Korea.

[3]National Park Institute of Wildlife Conservation, Gurye, 57616, Republic of Korea.

*Correspondence: Geun-Shik Lee, D. V. M., Ph. D.

Laboratory of Inflammatory Diseases, Department of Physiology, College of Veterinary Medicine, Kangwon National University, Chuncheon, Gangwon, 24341, Republic of Korea.

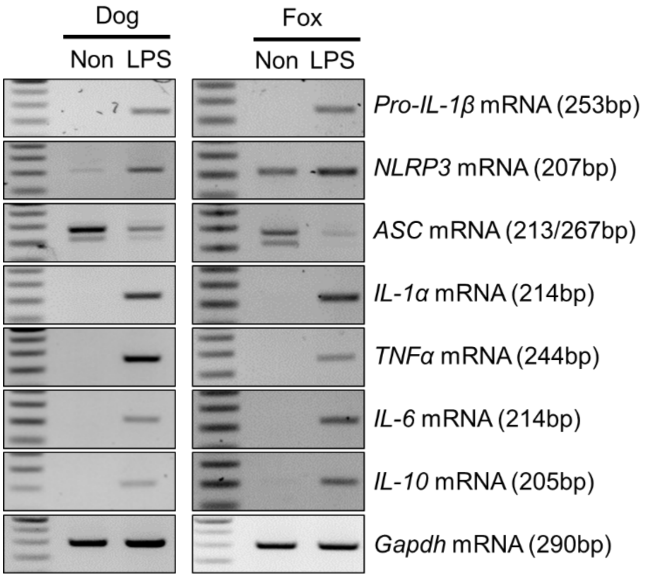

Supplementary Figure S1. Comparison of the gene expression of inflammasome components and inflammatory cytokines between dog and fox PBMCs.

Dog and fox PBMCs (2×10^6 cells per well in 12-well-plate) were treated with 1 μg/ml of LPS for 3 h, and the indicated gene expressions were analyzed by reverse transcription-polymerase chain reaction (RT-PCR). Briefly, total RNA isolated using NucleoZOL (MACHEREY-NAGEL GmbH & Co. KG, Postfach, Düren, Germany) and complementary DNA (cDNA) were synthesized using M-MLV reverse transcriptase and a random primer (Enzynomics Co., Daejeon, Republic of Korea). Genes were amplified by nTaq polymerase (Enzynomics), a SimpliAmp Thermal Cycler (Thermo Fisher Scientific), and gene-specific primers (**Supplementary Table S1**). The PCR products were analyzed by agarose gel electrophoresis and ethidium bromide staining.

PBMC: Peripheral blood mononuclear cells; LPS: Lipopolysaccharide; IL: Interleukin; TNFα: Tumor necrosis factor alpha; Pro-IL-1β: Pro-Interleukin-1 β; NLRP3: Nucleotide-binding oligomerization domain, leucine rich repeat and pyrin domain containing 3; ASC: Caspase recruitment domain; GAPDH: Glyceraldehyde-3-phosphate dehydrogenase.

Supplementary Table S1. Primers

Gene Name	Species	GenBank ID	Direction	Sequence (5' to 3')	Expected band size (bp)
Pro-IL-1β	Dog	NM_001037971	F	CTG TGG TCT TGG GCA TCA AA	253
			R	TGG CCA CCT CTG GTA TTT CC	
NLRP3	Dog	XM_005623149	F	ACT CTG TGA GGG CCT CTT GC	207
			R	AGG ATG CAG CCC TGT TGT TT	
ASC (pycard)	Dog	XM_014114362	F	CCT ATG GAC GCC ATG GAT CT	213, 267
			R	ATC CAG CAC CTC ATC CAC CT	
IL-1α	Dog	NM_001003157	F	TGG CCA AAG TTC CTG ACC TC	214
			R	GCT GCC ACC ACT ACC ACA TT	
TNFα	Dog	NM_001003244	F	GCT GCA CTT TGG AGT GAT CG	244
			R	CAA CCC ATC TGA CGG CAC TA	
IL-6	Dog	NM_001003301	F	CCA AGG ATG ATG CCA CTT CA	214
			R	TTG AAC CCA GAT TGG AAG CA	
IL-10	Dog	NM_001003077	F	TGG CTC AGC ACT GCT CTG TT	205
			R	GGA CCC GGT CAG CAG TAT GT	
GAPDH	Dog	NM_001003142	F	GCC ATC AAT GAC CCC TTC AT	290
			R	GCA TCA GCA GAA GGA GCA GA	

Pro-IL-1β: Pro-Interleukin-1 β; NLRP3: Nucleotide-binding oligomerization domain, leucine rich repeat and pyrin domain containing 3; ASC: Caspase recruitment domain; IL-1α: Interleukin-1α; TNFα: Tumor necrosis factor α; IL-6: Interleukin-6; IL-10: Interleukin-10, GAPDH: Glyceraldehyde-3-phosphate dehydrogenase

Supplementary Data S1. The sequence comparison of *pro-IL-1β* mRNA between dogs (GenBank ID, NM_001037971) and red foxes (XM_025994662)

```
Dog      1 atggcagcagtacccgaactcaccagtgaaatgatggcttactccagtaa   50
           ||||||||||||||||||||||||||||||||||||||||||||||||||
Fox      1 atggcagcagtacccgaactcaccagtgaaatgatggcttactccagtaa   50

Dog     51 caatgagaatgacctattctttgaagctgatggccctggaaatgtgaagt  100
           |||||||||||||.||||||||||||||||||||||||||||.|||||||
Fox     51 caatgagaatgacgtattctttgaagctgatggccctggaaacgtgaagt  100

Dog    101 gctgctgccaagacctgaaccacagttctctggtagatgagggcatccag  150
           ||||||||||||||||||||||||||||||||||||||||||||||||||
Fox    101 gctgctgccaagacctgaaccacagttctctggtagatgagggcatccag  150

Dog    151 ttgcaagtctcccaccagctctgtaacaagagtctgaggcatttcgtgtc  200
           ||||||||||||||||||||||||||||||||||||||||||||||||||
Fox    151 ttgcaagtctcccaccagctctgtaacaagagtctgaggcatttcgtgtc  200

Dog    201 agtcattgtagctttggagaagctgaagaagccctgcccacaggtcctcc  250
           ||||||||||||||||||||||||||||||||||||||||||||||||||
Fox    201 agtcattgtagctttggagaagctgaagaagccctgcccacaggtcctcc  250

Dog    251 aggaggatgacctgaagagcatcttttgctacatctttgaagaagaacct  300
           |||.||||||||||||||||||||||.|||||||||||||||||||||||
Fox    251 agggggatgacctgaagagcatctttcgctacatctttgaagaagaacct  300

Dog    301 atcatctgcaaaacagatgcggataattttatgagtgatgcagccatgca  350
           ||||||||||||||||||||.||||||||||||||||||||||||.|||.
Fox    301 atcatctgcaaaacagatgcagataattttatgagtgatgcagccctgcc  350

Dog    351 atcggtggactgcaagttacaggacataagccacaaatacctggtgctgt  400
           ||||||||||||||.||||||||||||||.||||||||||||||||||||
Fox    351 atcggtggactgcacgttacaggacataaaccacaaatacctggtgctgt  400

Dog    401 ctaactcatatgagcttcgggctctccacctcaatggggaaaatgtgaac  450
           |||||||.||||||||||||||||||||||||||||||||||||||||||
Fox    401 ctaactcgtatgagcttcgggctctccacctcaatggggaaaatgtgaac  450

Dog    451 aaacaagtggtgttccacatgagctttgtgcacggggatgaaagtaataa  500
           |||||||||||||||||||||||||||||||||||.||||||||||||.|
Fox    451 aaacaagtggtgttccacatgagctttgtgcacggagatgaaagtaatta  500

Dog    501 caagatacctgtggtcttgggcatcaaacaaaagaatctgtacctgtcct  550
           ||||||||||||||||||||||||||||||||||||||||||||||||||
Fox    501 caagatacctgtggtcttgggcatcaaacaaaagaatctgtacctgtcct  550

Dog    551 gtgtgatgaaggatggaaagcccaccctacagctagagaaggtagacccc  600
           ||||||||||||||||.|||||||||||||||||||||||||||||||||
Fox    551 gtgtgatgaaggatgggaagcccaccctacagctagagaaggtagacccc  600

Dog    601 aaagtctacccaaagaggaagatggaaaagcgatttgtcttcaacaagat  650
           ||||||||||||||||||||||||||||||||||||||||||||||||||
Fox    601 aaagtctacccaaagaggaagatggaaaagcgatttgtcttcaacaagat  650

Dog    651 agaaatcaagaacacagtggaatttgagtcttctcagtaccctaactggt  700
           ||||||||||||||||||||||||||||||||||||||||||||||||||
Fox    651 agaaatcaagaacacagtggaatttgagtcttctcagtaccctaactggt  700

Dog    701 acatcagcacctctcaagtcgaaggaatgcctgtcttcctaggaaatacc  750
           ||||||||||||||||||||||||||||||||||||||||||||||||||
Fox    701 acatcagcacctctcaagtcgaaggaatgcctgtcttcctaggaaatacc  750

Dog    751 agaggtggccaggatataactgacttcaccatggaattctcttcctag    798
           |.||||||||||||||||||||||||||||||||||||||||||||||
Fox    751 aaaggtggccaggatataactgacttcaccatggaattctcttcctag    798
```

Supplementary Data S2. The sequence comparison of pro-IL-1β amino acids between dogs (GenBank ID, NM_001037971) and red foxes (XM_025994662)

```
Dog                1 MAAVPELTSEMMAYSSNNENDLFFEADGPGNVKCCCQDLNHSSLVDEGIQ     50
                     |||||||||||||||||||||:||||||||||||||||||||||||||||
Fox                1 MAAVPELTSEMMAYSSNNENDVFFEADGPGNVKCCCQDLNHSSLVDEGIQ     50

Dog               51 LQVSHQLCNKSLRHFVSVIVALEKLKKPCPQVLQEDDLKSIFCYIFEEEP    100
                     ||||||||||||||||||||||||||||||||||.|||||||.|||||||
Fox               51 LQVSHQLCNKSLRHFVSVIVALEKLKKPCPQVLQGDDLKSIFRYIFEEEP    100

Dog              101 IICKTDADNFMSDAAMQSVDCKLQDISHKYLVLSNSYELRALHLNGENVN    150
                     |||||||||||||||:.||||.||||:|||||||||||||||||||||||
Fox              101 IICKTDADNFMSDAALPSVDCTLQDINHKYLVLSNSYELRALHLNGENVN    150

Dog              151 KQVVFHMSFVHGDESNNKIPVVLGIKQKNLYLSCVMKDGKPTLQLEKVDP    200
                     ||||||||||||||||.|||||||||||||||||||||||||||||||||
Fox              151 KQVVFHMSFVHGDESNYKIPVVLGIKQKNLYLSCVMKDGKPTLQLEKVDP    200

Dog              201 KVYPKRKMEKRFVFNKIEIKNTVEFESSQYPNWYISTSQVEGMPVFLGNT    250
                     ||||||||||||||||||||||||||||||||||||||||||||||||||
Fox              201 KVYPKRKMEKRFVFNKIEIKNTVEFESSQYPNWYISTSQVEGMPVFLGNT    250

Dog              251 RGGQDITDFTMEFSS    265
                     :||||||||||||||
Fox              251 KGGQDITDFTMEFSS    265
```

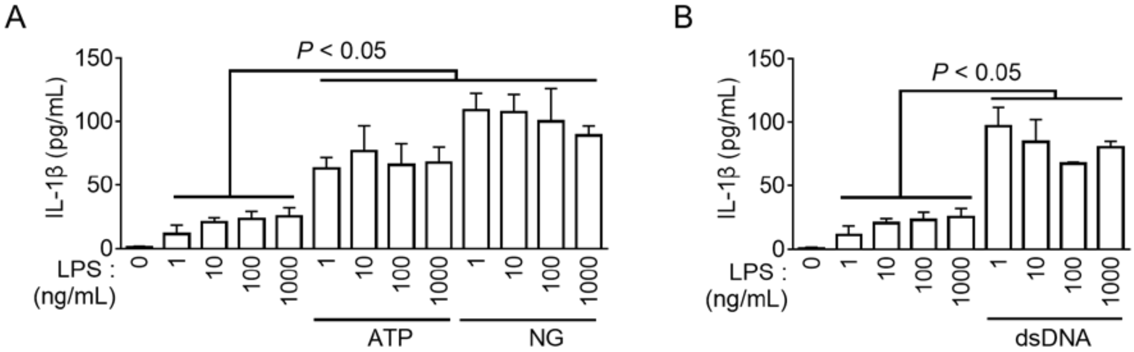

Supplementary Figure S2. The IL-1β secretion of fox inflammasome activation

Fox PBMCs were primed with LPS (1 to 1000 ng/mL) and then treated with NLRP3 inflammasome triggers (**A**, ATP, and NG) and an AIM2 trigger (**B**, dsDNA). The release of IL-1β was measured using ELISA. Bar graphs present the mean ± SD of at least three independent experiments.

IL-1β: Interleulin-1β; PBMC: Peripheral blood mononuclear cells; NLRP3: Nucleotide-binding oligomerization domain, leucine rich repeat and pyrin domain containing 3; ATP: Adenosine triphosphate; NG: Nigericin; AIM2: Absent in melanoma 2; dsDNA: Double-stranded DNA; ELISA: Enzyme-Linked Immunosorbent Assay; LPS: Lipopolysaccharide

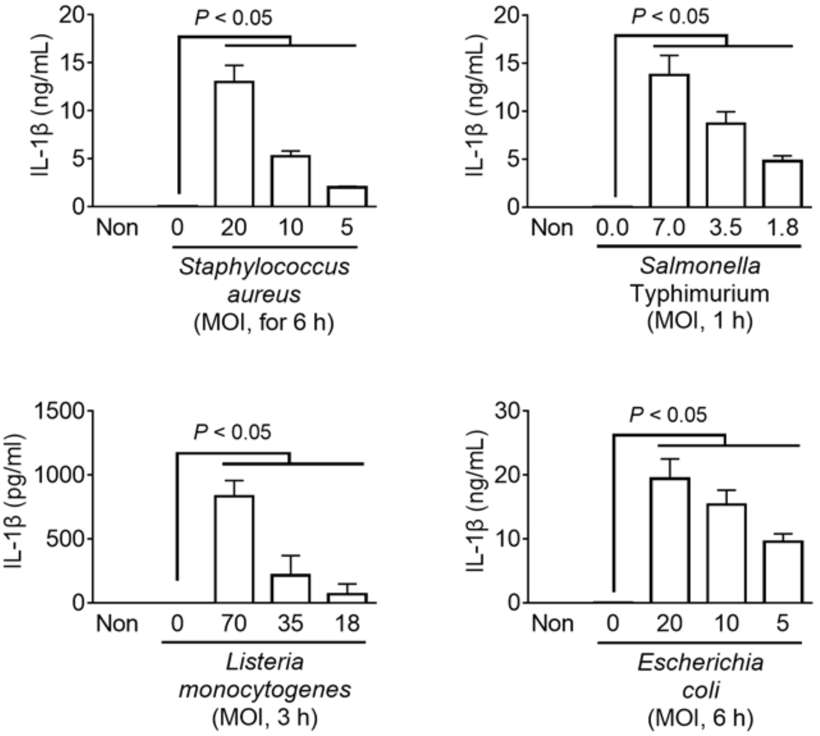

Supplementary Figure S3. Effect of bacteria on inflammasome activation in mouse BMDMs

LPS-primed BMDMs were treated with bacteria at the indicated multiplicity of infection (MOI) and the secretion of IL-1β was analyzed using ELISA. Bar graphs present the mean ± SD of at least three independent experiments.

LPS: Lipopolysaccharide; BMDM: Mouse bone marrow-derived macrophages; ELISA: Enzyme-Linked Immunosorbent Assay

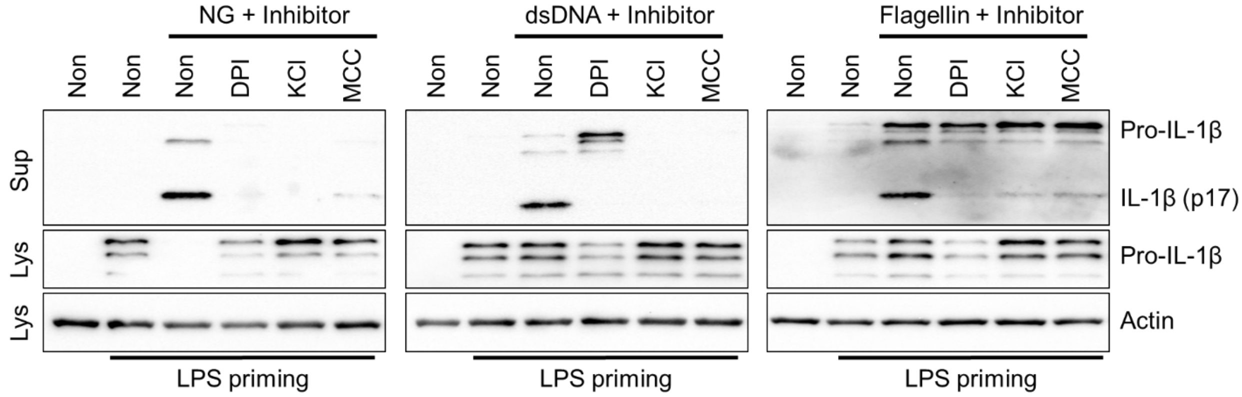

Supplementary Figure S4. Effect of Inhibitors on the fox inflammasome activation

LPS-primed fox PBMCs were treated with a ROS scavenger (diphenyleneiodonium [DPI]), a potassium efflux inhibitor (KCl), and an NLRP3 selective inhibitor (MCC950 [MCC]) in the presence of inflammasome triggers such as NG for NLRP3, dsDNA for AIM2, and flagellin for NLRC4. The secretion of IL-1β was analyzed by immunoblotting.

LPS: Lipopolysaccharide; ROS: Reactive oxygen species; NG: Nigericin; NLRP3: Nucleotide-binding oligomerization domain, leucine rich repeat and pyrin domain containing 3; dsDNA: Double-stranded DNA; AIM2: Absent in melanoma 2
